# Ethanolic Extract of Astragali Radix and Salviae Radix Prohibits Oxidative Brain Injury by Psycho-Emotional Stress in Whisker Removal Rat Model

**DOI:** 10.1371/journal.pone.0098329

**Published:** 2014-05-28

**Authors:** Hyeong-Geug Kim, Jin-Seok Lee, Min-Kyung Choi, Jong-Min Han, Chang-Gue Son

**Affiliations:** Liver and Immunology Research Center, Daejeon Oriental Hospital of Oriental Medical College of Daejeon University, Daejeon, Rep. of Korea; University of Modena and Reggio Emilia, Italy

## Abstract

Myelophil, an ethanolic extract of Astragali Radix and Salviae Radix, has been clinically used to treat chronic fatigue and stress related disorders in South Korea. In this study, we investigated the protective effects of Myelophil on a whisker removal-induced psycho-emotional stress model. SD rats were subjected to whisker removal after oral administration of Myelophil or ascorbic acid for consecutive 4 days. Whisker removal considerably increased total reactive oxygen species in serum levels as well as cerebral cortex and hippocampal regions in brain tissues. Lipidperoxidation levels were also increased in the cerebral cortex, hippocampus regions, and brain tissue injuries as shown in histopathology and immunohistochemistry. However, Myelophil significantly ameliorated these alterations, and depletion of glutathione contents in both cerebral cortex and hippocampus regions respectively. Serum levels of corticosterone and adrenaline were notably altered after whisker removal stress, whereas these abnormalities were significantly normalized by pre-treatment with Myelophil. The NF-κB was notably activated in both cerebral cortex and hippocampus after whisker removal stress, while it was efficiently blocked by pre-treatment with Myelophil. Myelophil also significantly normalizes alterations of tumor necrosis factor-α, interleukin (IL)-1β, IL-6 and interferon-γ in both gene expressions and protein levels. These results suggest that Myelophil has protective effects on brain damages in psycho-emotional stress, and the underlying mechanisms involve regulation of inflammatory proteins, especially NF-κB modulation.

## Introduction

Stress has been recognized as an inevitable phenomenon in the modern society. The excessive psychological and emotional stress has negatively affected both mental and physical health conditions [1]. Stress can directly evoke dysfunctions of brain activity, immune system, cardio-vascular, neuroendocrine, and central nervous and sympathetic nervous systems via hypothalamus-pituitary-adrenal (HPA) axis activation [2], [3]. Therefore, excessive and uncontrolled psycho-emotional stress is closely linked to the various spectrums of disorders, such as depression, anxiety, concentration disorders, cancer recurrence, cardiovascular diseases, and chronic fatigue syndrome [4]–[6].

Brain is the main target organ under uncontrolled stress status, and it is related with stress hormones and oxidative stress [7]. HPA-axis activation leads to excessive secrete stress hormones such as corticosterone (class of glucocorticoids) and catecholamine (adrenaline, nor-adrenalin and dopamine) [8], [9].

The high concentration of stress hormones, especially corticosterone, not only lead to damage brain cells (hippocampus, amygdale and cerebral cortex), but also accelerate neurodegenerative disorders of brain [10]. The high level of corticosterone induces oxidative stress via production of excessive reactive oxygen species (ROS) [9]. Additionally brain tissue is more susceptible to oxidative stress damages than other organs [11]. Therefore the over secretion of corticosterone, as a final product of stress response, is believed to damage brain cells due to oxidative stress [12]. Thus regulation of stress response via HPA-axis activation and antioxidant therapies has been a strategy in therapeutics for stress-associated CNS injuries [13].

Myelophil, (30% ethanolic extracts of the Astragali Radix and Salviae Radix), is a commercially available supplement and has been used for patients with chronic fatigue-associated disorders including concentration deficit or lack of memory functions in South Korea [14]. Our previous studies revealed that Myelophil has the potent antioxidant effect on oxidative brain injury in physical-dominant stress conditions including chronic restraint and cold stress model [15], [16]. Meanwhile, psycho-emotional stress happens more frequently in modern society [17], [18].

The present study is aim to investigate the brain-protective action focused on cerebral cortex and hippocampus and its underlying mechanisms using psycho-emotional stress model by whisker removal of rats.

## Materials and Methods

### Preparation of Myelophil and fingerprint analysis

Myelophil contains equal amounts of the herbs Astragali Radix (Astragalus membranaceus) and Salviae Radix (Salvia miltiorrhiza). All herbs used in this formulation complied with Korean Pharmacopoeia standards. Myelophil was manufactured by Kyung-Bang Pharmacy (Incheon, Rep. of Korea), according to the approved good manufacturing practice (GMP) guidelines of the Korean Food and Drug Administration (KFDA), according to over-the-counter Korean monographs. Briefly, 100 kg of Myelophil was boiled in 1000 L of 30% ethanol for 4 h at 100°C, and was then filtered using a 300-mesh filter (50 mm). Some parts were filtered through filter paper (Advantec, Toyo Roshi Kaisha, Tokyo, Japan) and lyophilized in our laboratory for this study. The final Myelophil product [yield 20.52% (w/w)] was stored for future use (VS No. KB-MYP-2011-01).

**Figure 1:**
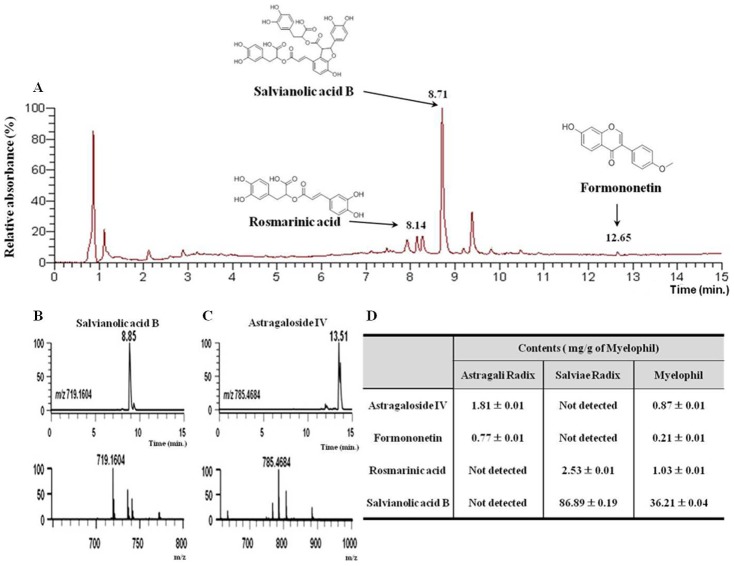
Fingerprinting analysis of Myelophil. Myelophil and three reference standards were subjected to ultra-high-performance liquid chromatography (UHPLC) analysis (A), Salvianolic acid B (m/z 719.1604, [M+H]^+^) (B) and (C) astragaloside IV (m/z 785.4684, [M+H]^+^), are the two major compounds in the qualitative histogram. Standards are shown in both the mass chromatogram and high-resolution mass spectra, and quantitative analysis of Myelophil (D).

The reproducibility of the Myelophil was confirmed by fingerprint using four reference compounds: astragaloside IV and formononetin for Astragali Radix, and salvianolic acid B and rosmarinic acid for Salviae Miltiorrhizae Radix, respectively. 20 mg of Myelophil and 10 µg of each reference compound were dissolved in 1 mL of 90% methanol, and the solution was filtered (0.45 µm). Samples and reference compound solutions were subjected to ultra-high-performance liquid chromatography-mass spectrometric analysis (UHPLC-MS) using an LTQ Orbitrap XL linear ion-trap MS system (Thermo Scientific Co., San Jose, CA, USA) equipped with an electrospray ionization source. Separation was performed using an Acquity BEH C18 column (1.7 µm, 100×2.1 mm; Waters, Milford, MA, USA) eluted at a flow rate of 0.3 mL/min using (0.1% formic acid in water) and (0.1% formic acid in acetonitrile) as mobile phases A and B, respectively, following the gradient program of : 0 to 1 min, 10% B (isocratic); 1 to 10 min, 10 to 90% B (linear gradient); 10 to 12 min, 100% B (isocratic) (Fig. 1- A to C).

### Chemicals and reagents

The following reagents were purchased from Sigma (St. Louis, MO, USA): 1,1,3,3-tetraethoxypropane, *N*,*N*-diethyl-*p*-phenylendiamine (DEPPD), ferrous sulfate, trichloroacetic acid (TCA), 5,5′-dithio-*bis*-(2-nitrobenzoic acid) (DTNB), 1-chloro-2,4-dinitrobenzene, potassium phosphate, reduced glutathione (GSH), 4-amino-3-hydrazino-5-mercapto-1,2,4-triazole (Purpald), myoglobin, 2,2′-azino-*bis*(3-ethylbenzothiazoline-6-sulfonic acid) diammonium salt (ABTS), glutathione reductase (GSH-Rd), l-glutathione oxidized disodium salt (GSSG), β–NADPH, and tert-butyl hydroperoxide. Antibodies against 4-hydroxynonenal (4-HNE) and 3-amino-9-ethylcarbazole (AEC) were obtained Abcam (Cambridge, MA, USA). Thiobarbituric acid (TBA) was obtained from the Lancaster Co. (Lancashire, England). Hydrogen peroxide was purchased from Junsei Chemical Co., Ltd. (Tokyo, Japan) and *n*-butanol from J.T. Baker (Mexico City, Mexico), 1 M Tris-HCl solution (pH 7.4) and 500 mM ethylenediaminetetraacetic acid (EDTA) solution (pH 8.0) were purchased from Bioneer (Daejeon, Republic of Korea). The antibodies (IκBα, NF-κB and β-actin) were purchased from Santa Cruz Biotechnology Inc. (Santa Cruz, CA).

### Ethical statement for experimental animals

Sixty specific-pathogen-free male Sprague-Dawley (SD) rats (six-weeks old, 190–210 g) were purchased from Koatech (Gyeonggi-do, Rep. of Korea). The rats were acclimated in an environmentally controlled room on acryl transparent cages (1 cage for 5 animals) at 22±2°C, 55%±10% relative humidity, and adopting 12 h light/dark cycle. The rats were fed commercial pellets (Gyeonggi-do, Rep. of Korea) and tap water *ad libitum* for one week. This experimental protocol was strictly designed and performed in accordance to the Guide for the Care and Use of Laboratory Animals (NIH publication No. 85–23, revised 1985), and approved by the Institutional Animal Care and Use Committee of Daejeon University (IAEC No: DJUARB 2013-003).

### Study design and experimental procedures

**Figure 2:**
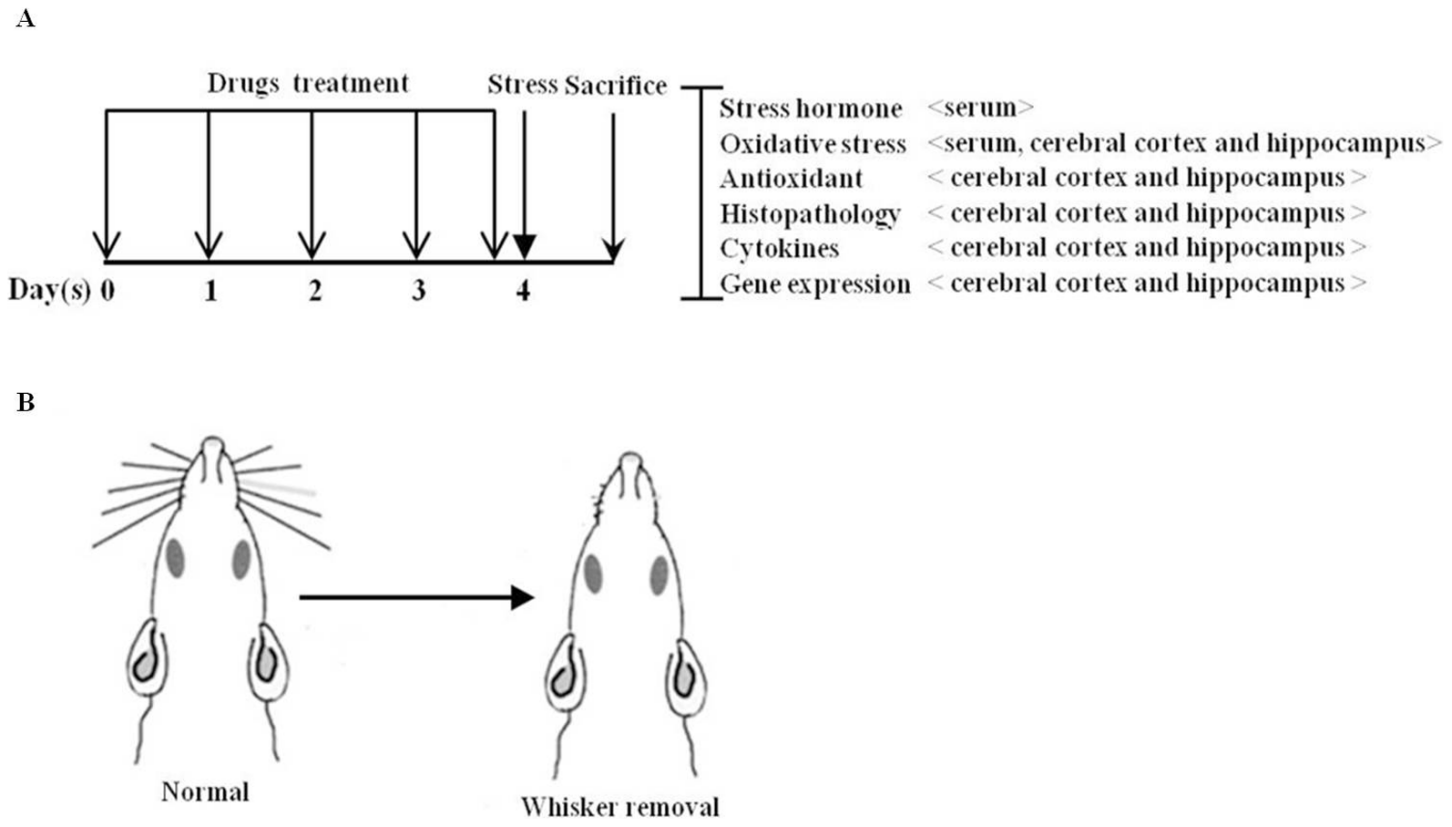
Experimental design and way to remove whisker of animals. Experimental schedule (A), and for induction psychological stress, the whiskers around the nose and mouth were completely cut off with scissors without the use of anesthesia (B). A total sprague sawley rats (n = 60) were treated with distilled water, Myelophil (50, 100 or 200 mg/kg, p.o) or (ascorbic acid 100 mg/kg, p.o) were orally administrated for consecutive 4 days. The last administrations of drugs were additionally administrated 2 hours before whisker removal stress without normal group. Twelve hours after, animals with whisker removal stress all animals were sacrificed under the ether anesthesia. After sacrificed the whole blood and brain tissues were isolated from animals for further assays.

After 1-week acclimatization, all rats were divided randomly into six groups (n = 10/group) as follows: normal (no whisker removal and distilled water), control (whisker removal and distilled water), Myelophil treatment groups (whisker removal and 50, 100, or 200 mg/kg Myelophil), and the positive control group (whisker removal and ascorbic acid 100 mg/kg). The Myelophil and ascorbic acid were dissolved in distilled water. The distilled water, Myelophil, or ascorbic acid was orally administered for 4 consecutive days and the last administration was 2 hours before whisker removal (Fig. 2-A to B).

To induce psychological stress, the whiskers around the nose and mouth were completely cut off with scissors without the use of anesthesia. After completely whisker removal, the rats were kept in a small square cage with free access to food and water. After 12 hours, rats were sacrificed under ether anesthesia, and whole blood was obtained from the abdominal aorta. To give the similar stress during experiment process, normal group was kept on the hand for approximately 2 min without cutting process [18].

The blood was allowed to clot at room temperature, and then the separated serum sample was used for measuring total reactive oxygen species (ROS) and stress-related hormones, and the whole brain tissue was removed immediately after sacrifice and stored at −80°C in RNAlater (Ambion, Austin, TX, USA).

The total ROS concentration and stress hormones were measured using the serum samples from all of rats (n = 10 for each group). Among the all animals, 3 rats in each group were used for histopathological analysis of brain tissue. From the remaining 7 rats in each group, cerebral cortex and hippocampus were dissected. A part of each tissue was used for measuring oxidative stress parameters, and the other part was used for gene expression analysis (n = 4) and western blot analysis (n = 3) (Fig. 2-A).

### Brain dissections, tissue homogenization, and protein quantification

Discrete regions of the brain were dissected on an ice-cold glass plate [19]. The dissected brain tissues were homogenized in radio-immuno recipitation assay (RIPA) buffer for the enzyme activity analyses, and protein concentrations were determined using a bicinchoninic acid protein assay kit (St. Louis, MO, USA). Brain tissues were homogenized in 1.15% potassium chloride (w/v) for malondialdehyde (MDA) and protein carbonyl formation assays.

### Histopathological and immunohistochemical analyses

Prefrontal cortex and hippocampal *cornus ammonis* (CA) 1 regions were observed using hematoxylin and eosin (H&E) staining and 4-hydroxynonenal (4-HNE) staining. Brain tissues were fixed in 10% formalin embedded with paraffin for histopathological examination. H&E staining was performed according to standard procedures. Sections were incubated with 4-HNE (1∶50; Abcam, Cambridge, MA, USA) primary antibody and biotinylated secondary antibody (Nichirei Biosciences, Tokyo, Japan), followed by the avidin-biotin-peroxidase complex. The immunoreactive signal was developed using the AEC substrate. The slides were counterstained with hematoxylin (Sigma-Aldrich), examined under optical microscope (Leica Microsystems, Wetzlar, Germany). The neuronal cell layer areas in prefrontal cortex and hippocampal CA1 regions, 4-HNE positive signaling density in cerebral cortex and hippocampal CA1 regions were analyzed using Image J software (NIH, Bethsda, MD).

### Determination of total ROS levels

Total ROS in the serum and brain dissected tissue (cerebral cortex and hippocampus) levels were determined according to a previous method with simply modified using H_2_O_2_ to generate the standard calibration curve [20]. DEPPD and ferrous sulfate standard stock solutions were prepared. Standard/sample solution (3 µL) was transferred to a 96-well microplate, 183 µL of reagent mixture (3 µL of 6 mg/mL DEPPD and 180 µL of 4.37 µM ferrous sulfate in 0.1 M sodium acetate buffer, pH 4.8) was added, absorbance measured at 505 nm at 37°C using microplate reader (Molecular Device Corp., Sunnyvale, CA, USA).

### Determination of malondialdehyde (MDA) lipid peroxidation

Lipid peroxidation levels in brain tissue were determined according to a thiobarbituric acid reactive substances (TBARS) method described previously [21]. Briefly, brain tissues were homogenized using RIPA buffer, and 50 µL of the homogenate was mixed with 500 µL of 20% tri-chloroacetic acid and 250 µL of 0.67% TBA. The mixture was incubated at 100°C for 30 min, 1.03 mL *n*-butanol was added, vortexed and centrifuged at 3,000×*g* for 15 min at 4°C. Absorbance was measured at dual mode of 535 and 520 nm using UV Spectrophotometer. TBARS concentration was calculated with a 1,1,3,3-tetraethoxy-propane (TEP) standard curve and expressed as µM/mg protein.

### Determination of total antioxidant capacity (TAC), total glutathione (GSH) contents, superoxide dismutase (SOD) and catalase activity

Total antioxidant capacity (TAC) in cerebral cortex and hippocampus were determined according to previous method [22]. 10 mM phosphate-buffered saline (pH 7.2, 90 µL), 18 µM myoglobin solution (50 µL), 3 mM 2,2′-azino-bis (3-ethylbenzthiazoline-6-sulfonic acid) diammonium salt solution (20 µL), sample diluted homogenates sample (20 µL), and various concentrations of gallic acid were added to a 96-well microplate mixed well at 25°C for 3 min. Then, 20 µL of 2.5 mM hydrogenperoxide (H_2_O_2_) was added to each well and incubated for 5 min. The absorbance was measured using a plate reader (Molecular Device Corp., Sunnyvale, CA) at 600 nm. TAC was expressed as gallic acid equivalent antioxidant capacity.

Total glutathione content in the cerebral cortex and hippocampus were determined as described previously [23]. Briefly, dissected brain tissue was homogenized with 1 mL of RIPA buffer, centrifuged at 10,000×g for 15 min, 4°C. The supernatant was transferred to a clean tube, and duplicate samples of 50 µL homogenate diluted in PBS 10 mM, pH 7.2) or GSH standard were combined with 80 µL of DTNB/NADPH mixture (10 µL of 4 mM DTNB and 70 µL of 0.3 mM NADPH) in 96-well microplate. 20 µL (0.06 U) of GSH-reductase solution was added to each well, and the resulting absorbance was measured at 412 nm using a plate reader (Molecular Devices, Sunnyvale, CA).

### Determination of catalase and superoxide dismutase (SOD) activity

Catalase activities in cerebral cortex and hippocampus were assayed as described previously [24]. Briefly, 150 µL of phosphatase buffer (250 mM, pH 7.2),150 µL of 12 mM methanol and 30 mL of 44 mM H_2_O_2_ were mixed with 300 µL of each homogenate sample/standard solutions in a 13×100 mm test tube. The reaction was allowed to proceed for 10 to 20 min and was stopped by the addition of 450 mL of Purpald solution (22.8 mM of Purpald in 2 N potassiumhydroxide). The mixture was kept for 20 min at 25°C, followed by addition of 150 µL of potassium periodate (65.2 mM in 0.5 N potassiumhydrate). The absorbance of the purple formaldehyde adduct formed was measured at 550 nm using a spectrophotometer (Molecular Devices, Sunnyvale, CA).

SOD activity in cerebral cortex and hippocampus was determined using a SOD assay kit (Dojindo Laboratories, Kumamoto, Japan), according to the protocol from the manufacture. Bovine erythrocyte SOD (Sigma) was used as a standard (0.01 - 20 U/mL).

### Gene expression analysis using real-time polymerase chain reaction (PCR) in brain tissues

The dissected brain regions (cerebral cortex or hippocampus) were used to analyze the expression of 6 genes. Total RNA was extracted using TRIzol reagent (Molecular Research Center, Cincinnati, OH, USA), and used for cDNA synthesis with 2 mg of RNA and a High-Capacityc DNA Reverse Transcription kit (Ambion). The primer sequences were as follows: (forward and reverse, respectively) IL-1β for TCT GAC CCA TGT GAG CTG AAA G and CGT TGC TTG TCT CTC CTT GTA CA; IL-6 for TGA AAC CCT AGT TCA TAT CTT CAA ACA and CCA CTC CTT CTG TGA CTC TAA CTT CTC; TNF-α for ATG GGC TCC CTC TCA TCA GTT and CTC CGC TTG GTG GTT TGC TG; iNOS for GAA AGC GGT GTT CTT TGC TTC T and CGC TTC CGA CTT TCC TGT CT; IFN-γ for GAA AGA CAA CCA GGC CAT CAG and TGC TCA TGA ATG CAT CCT TTT T; IL-10 for GCC AAG CCT TGT CAG AAA TGA and TCC CAG GGA ATT CAA ATG CT; β-actin for CTA AGG CCA ACC GTG AAA AGA T and GAC CAG AGG CAT ACA GGG ACA A, respectively. Reactions were performed with 12.5 mL of iQ SYBR Green Supermix, 1 mL of 10 pM/L primer pair, 10.5 mL of distilled water, and 1 mL of cDNA. Each PCR was performed under the following conditions: 95°C for 5 min followed by 40 cycles of 95°C for1 min, 58°C for 40 s and 72°C for 40 s, followed by a single fluorescence measurement. For analysis of data, the gene expression levels were compared with those of β -actin as a reference gene.

### Western blot analysis

The brain tissues of each rat were extracted by cytoplasmic extracts (for IκBα and β-actin), and nuclear extracts (for NF-κB) fractionated by 10% sodium dodecyl sulfate-polyacrylamide gel electrophoresis (SDS-PAGE) and transferred onto a nitrocellulose membranes. Cytosolic extracts were prepared in hypotonic buffer 10 mM *N*-(2-hydroxyethyl)-piperazine-*N*-2-ethanesulfonic acid (HEPES, pH 7.6), 10 mM KCl, 2 mM MgCl_2_, 1 mM dithiothreitol, 0.1 mM ethylene diamine tetra acetic acid (EDTA), and 0.1 mM phenyl methyl sulfonyl fluoride (PMSF). Nuclear extracts were prepared in hypertonic buffer consisting of 50 mM HEPES (pH 7.9), 400 mM KCl, 0.1 mM EDTA, and 10% glycerol. Each transfer membrane was blocked overnight at 4°C with blocking solution [10 mM Tris–HCl (pH 7.4), 125 mM NaCl, 0.1% Tween 20, and 5% skim milk] incubated with specific antibodies for 1–3 h at room temperature. The blots were washed 3 times using washing buffer (20 mM Tris, 160 mM NaCl, and 0.1% Tween 20), followed by incubation for 1 hour with the appropriate horseradish peroxidase- conjugated secondary antibody. The peroxidase bound to the blot was detected using the Immobilon Western HRP detection reagent (Millipore, Billerica, MA, U.S.A.). The ratio of the protein interested was subjected to β-actin and was densitometrically analyzed by Image J software (NIH, Bethsda, MD).

### Statistical analysis

All data are expressed as the mean ± standard deviation (SD). Statistically significant differences between the groups were analyzed by one-way analysis of variance (ANOVA) followed by post hoc multiple comparison Fisher's LSD t-test using the IBM SPSS statistics 20.0 (SPSS Inc. Chicago, IL, USA). Differences at *p*<0.05, *p*<0.01, or *p*<0.001 were considered statistically significant.

## Results

### Effects on the oxidative stress parameters

Whisker removal stress considerably increases the total ROS approximately 1.4-, 1.4- and 2.2-folds higher than those in the normal groups in the serum, cortex and hippocampus, respectively, whereas the elevated ROS levels were significantly attenuated by pre-treatment with Myelophil as compared with control groups (*p*<0.001 for 50, 100 and 200 mg/kg in both serum and cerebral cortex, *p*<0.05 for 200 mg/kg in hippocampus, respectively, Table 1). The MDA concentrations were higher about 2.0- and 1.6 -fold than those in the normal group in cerebral cortex and hippoc_campus, respectively. Pre-treatment with Myelophil significantly attenuates the MDA levels in hippocampus regions (*p*<0.001 for 200 mg/kg in hippocampus, Table 1), but the cerebral cortex MDA levels were slightly decreased by pre-treatment with Myelophil without statistical significance. The ascorbic acid also showed the similar effects on the MDA concentrations as well as total ROS levels (Table 1).

**Table 1:** Changes of oxidative stress and antioxidant system components.

Groups	Region	Normal	Control	MYP 50	MYP 100	MYP 200	Vita C 100
MDA contents	Cerebral cortex	19.7±3.25	39.1±4.7#	29.4±13.8	35.6±5.1	29.7±7.0	21.6±14.1***
(µM/mg protein)	Hippocampus	3.6±0.2	4.4±0.9#	3.8±0.5	4.3±0.3	2.2±0.5***	4.2±0.7
Total ROS	Serum	138.7±6.6	207.2±71.1###	135.1±9.9***	141.1±11.7***	138.1±15.1***	160.3±12.3**
(units/mL serum or	Cerebral cortex	264.3±41.8	375.3±6.9###	295.6±20.3***	227.6±37.3***	189.6±22.2***	185.2±23.4***
mg protein)	Hippocampus	325.3±147.3	730.9±379.8#	636.7±489.2	656.6±249.6	271.9±10.0*	624.3±374.8
TAC	Cerebral cortex	706.339.2	706.6±54.2	774.7±54.5	743.6±106.8	845.8±63.6**	927.0±36.7***
(µM/mg protein)	Hippocampus	424.7±38.1	413.3±40.6	400.4±23.6	427.1±56.3	515.7±17.4***	609.3±19.5***
Total GSH	Cerebral cortex	493.2±78.0	372.7±109.0#	608.7±94.4***	566.9±134.0***	522.9±104.8**	269.9±107.5
(uM/mg protein)	Hippocampus	147.5±9.8	117.8±12.4#	190.5±31.9***	237.5±16.3***	522.3±32.2***	192.7±21.5***
SOD activity	Cerebral cortex	28.7±8.1	32.5±9.6#	48.0±16.7*	53.9±15.0**	46.0±10.1	26.8±12.3
(U/mg protein)	Hippocampus	19.7±1.9	23.0±2.1	27.7±3.8*	32.2±2.9***	29.8±2.2**	22.7±3.5.
CAT activity	Cerebral cortex	50.4±37.5	98.3±45.6	164.9±56.8	151.0±74.3	137.7±16.1	116.6±80.2
(U/mg protein)	Hippocampus	117.8±23.5	153.3±30.7#	146.2±12.7	178.7±16.2	174.7±8.6	97.5±30.9**

Data were expressed as the mean ± SD. #p<0.05, ##p<0.01 and ###p<0.001compared with the normal group; *p<0.05, **p<0.01 and ***p<0.001 compared with the control group. CAT, catalase; GSH, glutathione; ROS, reactive oxygen species; SOD, superoxide dismutase; TAC, total antioxidant capacity.

### Effects on the histopathological aspects

**Figure 3:**
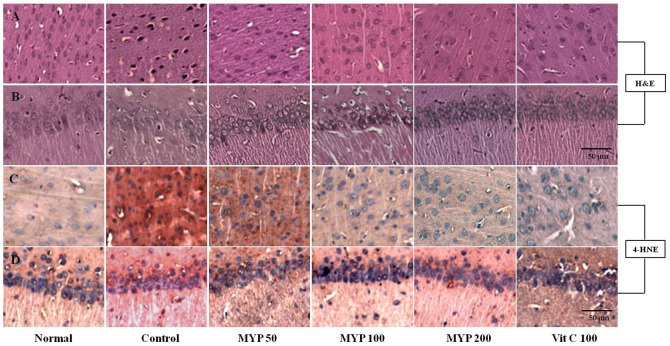
Histopathological and immunohistochemistry findings. Hematoxylin and eosin (H&E) staining in prefrontal cortex (A) and hippocampal CA1 regions (B). The immunohistochemistry for 4-hydroxynonenal (4-HNE) were performed in prefrontal cortex (C) and hippocampal CA1 regions (D). The slides were observed by light microscopy (200× magnification, n = 3). The reference bar indicated 50 µm.

Whisker removal stress induces histological alterations in both cerebral cortex and hippocampus, respectively. The cells were condensed and shrunken in cerebral cortex and these alterations were also observed in hippocampal region, especially CA1 area. However pre-treatment with Myelophil efficiently blocked these alterations as shown by H&E staining compared with control groups, respectively (Fig. 3-A and B, Fig. S1-A and B). Immunostaining results reveals that, 4-HNE signals were considerably increased in both cerebral cortex and hippocampal CA1 region (parts of red-brown color) after whisker removal stress compared with normal group, and these alterations was remarkably ameliorated by pre-treatment with Myelophil (Fig. 3-C and D, Fig. S1-C and D). The cell death signaling was remarkably increased in both prefrontal cortex and hippocampal CA1 region (part of red color) by whisker removal stress as compared with normal group. Pre-treatment with Myelophil efficiently attenuated those alterations both cerebral cortex and hippocampal CA1 region (Fig. S2-A and B). Ascorbic acid also slightly improved the histopathological and immunohistochemistry findings (Fig. 3-A to D, Fig. S1-A to D, Fig. S2-A and B).

### Effects on the TAC and GSH in brain tissue

Whisker removal stress didn't alter the TAC levels in brain cerebral cortex and hippocampus compared with those in normal groups. However, pre-treatment of Myelophil significantly increased the TAC levels in hippocampus compared with those in the control groups (*p*<0.01 for 200 mg/kg in cerebral cortex, *p*<0.001 for 200 mg/kg in hippocampus, respectively, Table 1). Total GSH contents were considerably depleted by 0.7-and 0.8-fold following whisker removal stress compared with those in the normal groups in cerebral cortex and hippocampus, respectively. Pre-treatment with Myelophil significantly restored the depleted total GSH contents in brain tissues (*p*<0.01 for 200 mg/kg, *p*<0.001 for 50 and 100 mg/kg in cerebral cortex; for 50, 100 and 200 mg/kg in hippocampus, respectively, Table 1). Pre-treatment with ascorbic acid positively affected TAC levels in both cerebral cortex and hippocampus, and it also beneficially worked on the total GSH contents in hippocampus (Table 1)

### Effects on the SOD and catalase activities in brain tissue

Whisker removal stress significantly increased the activities of SOD in hippocampus by 1.2-fold (*p*<0.05), but not cerebral cortex (about 1.2-fold changes) as compared with those in the normal groups, respectively. Pre-treatment with Myelophil caused significant increases of SOD activities as compared with control groups (*p*<0.05 for 50 mg/kg, *p*<0.01 for 100 mg/kg in cerebral cortex; *p*<0.05 for 50 mg/kg, *p*<0.01 for 200 mg/kg, *p*<0.001 for 100 mg/kg in hippocampus, respectively, Table 1). The catalase activities were also significantly increased in hippocampus about 1.3-fold (*p*<0.05), and these enzymes were slightly higher in cerebral cortex by 1.3-fold (*p*>0.05) following whisker removal stress as compared with those in normal groups. Pre-treatment with Myelophil more slightly increased the enzyme activities as compared with control groups without statistical significances (Table 1). Ascorbic acid treatment didn't affect both the SOD and catalase activities (Table 1).

### Effects on the serum levels of corticosterone and adrenaline

**Figure 4:**
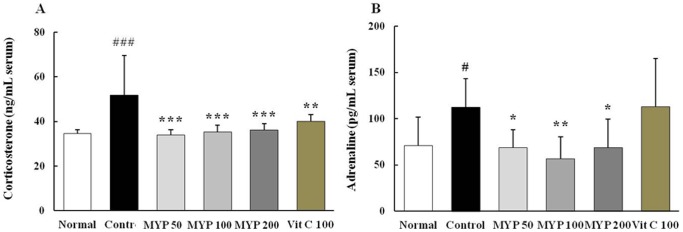
Serum levels of stress-associated hormones. Serum levels of corticosterone (A) and adrenaline (B) were determined by enzyme-linked immunosorbent assay. Data are means ± standard deviations (n = 10). ^#^ *p*<0.05 compared with the normal group; ^*^ *p*<0.05 and ^**^ *p*<0.01 compared with the control group.

Whisker removal stress caused 1.5-fold increases in serum corticosterone levels compared with normal group, whereas Myelophil administration significantly decreases these levels compared with control group (*p*<0.001 for 50, 100 and 200 mg/kg, Fig. 4-A). Adrenaline in the serum levels were also increased approximately 1.5-fold following whisker removal stress in comparison to normal group, while pre-treatment with Myelophil significantly reduces adrenaline in the serum levels compared with control group (*p*<0.05 for 50 and 200 mg/kg, *p*<0.01 for 100 mg/kg, Fig. 4-B). Ascorbic acid tended to have some effects on changes in the serum corticosterone levels, but not adrenaline levels (*p*<0.01, Fig. 4-A).

### Effects on the inflammatory cytokines in brain tissue

**Figure 5:**
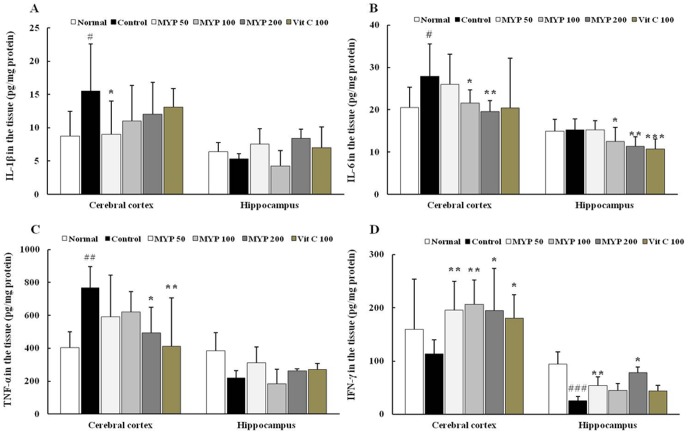
Inflammatory and immune-related cytokine levels in brain tissues. Protein levels of interleukin (IL)-1β (A), tumor necrosis factor-α (TNF-α) (B), IL-6 (C) and interferone-γ (IFN-γ) (D) were determined by enzyme-linked immunosorbent assay in cerebral cortex and hippocampus, respectively. Data are means ± standard deviations (n = 7). ^#^ *p*<0.05 and ^##^ *p*<0.01compared with the normal group; ^*^ *p*<0.05 and ^**^ *p*<0.01compared with the control group.

Whisker removal stress significantly increases IL-1β, IL-6 and TNF-α protein levels in cerebral cortex by 1.8-, 1.4- and 1.9-folds in comparison with those in the normal groups, whereas pre-treatment with Myelophil significantly attenuated these elevated cytokine levels compared with control groups (*p*<0.05 for 50 mg/kg in IL-1β, for 100 mg/kg in IL-6, for 200 mg/kg in TNF-α; *p*<0.01 for 200 mg/kg in IL-6, Fig. 5-A to C). These cytokines were not drastically affected by whisker removal in hippocampus regions, whereas pre-treatment with Myelophil significantly decreased the protein levels IL-6 compared with control group (*p*<0.05 for 50 mg/kg and *p*<0.01 for 100 mg/kg, Fig. 5-B). The protein levels of IFN-γ in cerebral cortex and hippocampus were considerably decreased about 0.7- and 0.3-fold compared with those in the normal groups, and pre-treatment with Myelophil significantly recovered the depletion of IFN-γ levels by whisker removal (*p*<0.05 for 200 mg/kg in cerebral cortex and hippocampus; *p*<0.01 for 50 and 100 mg/kg in cerebral cortex, for 200 mg/kg in hippocampus respectively, Fig. 5-D). Administration of ascorbic acid only attenuates the IL-1β levels (Fig. 5-A).

### Effects on the mRNA expressions in brain tissue

**Figure 6:**
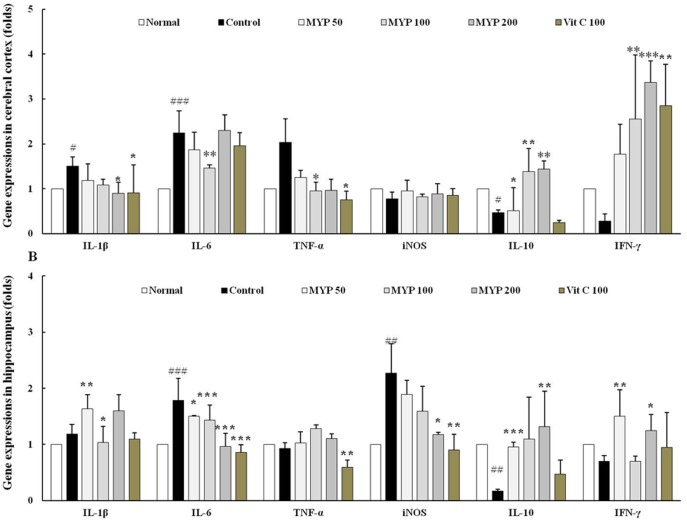
Gene expression levels of inflammatory and immune-related cytokines in brain tissues. Gene expression levels of interleukin (IL)-1β, tumor necrosis factor-α (TNF-α), IL-6, inducible nitric oxide synthase (iNOS), IL-10 and interferone-γ (IFN-γ) (D) were determined using real-time PCR. Data are means ± standard deviations (n = 4). ^#^ *p*<0.05, ^##^ *p*<0.01, and ^###^ *p*<0.001 compared with the normal group; ^*^ *p*<0.05 and ^***^ *p*<0.001 compared with the control group.

Whisker removal stress considerably up-regulates the gene expressions of inflammatory reaction related genes. The gene expression levels of IL-1β, IL-6 and TNF-α were approximately 1.5-, 2.2-and 2.0-fold higher than those in the normal groups, and the IL-10 and IFN-γ were down-regulated about 0.5- and 0.3-fold by whisker removal in cerebral cortex. Myelophil pre-treatment significantly attenuated these alterations similar to normal levels as compared with control group (*p*<0.05 for 200 mg/kg in IL-1β, for 100 mg/kg in TNF-α, for 50 mg/kg in IL-10; *p*<0.01 for 100 mg/kg in IL-6 and IFN-γ, for 100 and 200 mg/kg in IL-10; *p*<0.001 for 200 mg/kg in IFN-γ, respectively, Fig. 6-A). In the hippocampus, the gene expression levels of IL-1β, IL-6 and iNOS were about 1.2-, 1.8-and 2.3-fold times compared with the normal groups, and the IL-10 and IFN-γ were down-regulated about 0.2-and 0.7-fold by whisker removal in hippocampus, concurrently. Pre-treatment with Myelophil significantly exerted to normalize these alterations (*p*<0.05 for 100 mg/kg in IL-1β, for 50 mg/kg in IL-6, and for 200 mg/kg in iNOS; *p*<0.01 for 50 mg/kg in IL-1β and IFN-γ, for 200 mg/kg in IL-10; *p*<0.001 for 100 and 200 mg/kg in IL-6, for 50 mg/kg in IL-10, respectively, Fig. 6-B). Ascorbic acid treatment shows similar results to those of Myelophil (Fig. 6-A and B).

### Effects on NF-κB activation in brain dissected tissue

**Figure 7:**
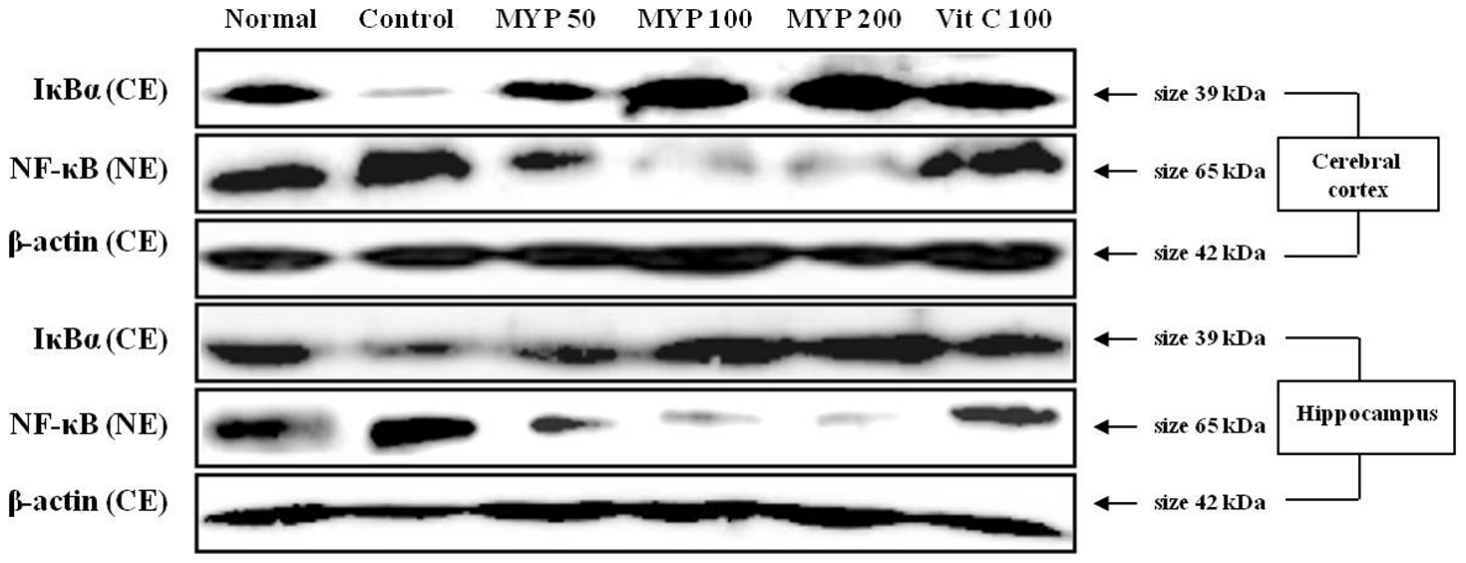
Western blot analysis of NF-κB activation in brain tissues. IκBα (cytosolic extract) (A) and NF-κB (nuclear extract) (B) were detected by their specific antibodies (n = 3).

Whisker removal stress considerably increases cytosolic IκBα degradation in both cerebral cortex and hippocampus. Pre-treatment with Myelophil remarkably blocks the degradation of cytosolic IκBα in both cerebral cortex and hippocampus (Fig. 7). NF-κB notably induces the translocation into the nucleus of both cerebral cortex and hippocampus by whisker removal stress, while pre-treatment with Myelophil efficiently decreases NF-κB translocation compared with control groups (Fig. 7, Fig. S3-A to D). Ascorbic acid positively showed the similar effects of Myelophil (Fig. 7 and Fig. S3-A to D).

## Discussion

It is well documented that both hypothalamus-pituitary-adrenal (HPA) axis and sympatho-adrenal-medullary (SAM) axis were the main routes for the stress responses. Maladaptation of stress response systematically affects all aspects of our body and mind through abnormal HPA- and SAM-axis activation [25]. The psycho-emotional stress has been recognized as one of pivotal medical issue owing to its deeply linkage to various diseases including vascular disorders, stroke, hepatic disorders and cancer occurrences [26].

Among the different stress animal models, such as immobilization, electric shock, forced swimming and cold stress models, the whisker removal model is a typical method to mimic a psycho-emotional stress in animal model [17], [18]. In the present study, whisker removal causes considerable damages to brain tissues of both cerebral cortex and hippocampus as shown by the histological findings (Fig. 3-A and B, Fig. S1-A and B). Whiskers play key roles as a locomotive sensor in rodents. The sensory input is directly connected to motor neurons organizing the locomotive activities [27]. Therefore the whisker removal not only leads to anxiety-associated stress behavior [17], [18], but also induces the oxidative stress in brain tissue, which were evidenced by elevation of total ROS levels and MDA contents (Table 1). The excess 4-HNE signals, which are produced by lipid peroxidation in cells, were observed at the same regions after whisker removal stress (Fig. 3-C and D, Fig. S1-C and D). Excessive formation of ROS in the brain tissues can evoke the neurodegenerative diseases such as Alzheimer's disease as well as Parkinson's diseases [28].

In particular, brain has been known to be vulnerable organs to oxidative stress damage due to its high rate of oxygen consumptions, plenty of polyunsaturated fatty acids and metal irons transition, as well as high sensitivity of blood-brain endothelial cells [11], [29]. Among brain regions, prefrontal cortex, hippocampus and amygdala were known to be more susceptible to oxidative stress than other regions of brain tissue [30]. The cell death and thin layer in both prefrontal cortex and hippocampal CA 1 regions were resulted from oxidative stress, evidenced by 4-HNE staining (Fig. S2-A to B, Fig. 3-C and D, Fig. S1-C and D).

We pre-treated Myelophil for 4-consecutive days before whisker removal, the brain tissue damages were significantly attenuated in comparison with control group (Fig. 3-A to D, Fig. S1-A to D, Fig. S2-A and B). These histopathological findings were in accordance with biomarkers of oxidative and antioxidant components of brain tissues (Table 1). Accumulated studies reported that the linkage between emotional stress and oxidative brain injury [17], [18]. Whisker removal stress systemically and significantly induced the imbalance between oxidative stress and antioxidant components. However, the pre-treatment with Myelophil significantly attenuates these alterations (Table 1). Interestingly, two major antioxidant enzymes; SOD and catalase, activities were significantly higher than those of normal groups after whisker removal stress, and these activities were further augmented by pre-treatment with Myelophil in comparison to control group. These phenomena might come from the compensating response against whisker removal-induced oxidative stress.

As our expectation, two major stress hormones, corticosterone and adrenaline, were notably increased in the serum levels approximately by 1.5-and 1.6-fold, respectively, compared with normal group (Fig. 4-A and B). Corticosterone is released from adrenal cortex, and this stress hormone activates the synthesis of adrenaline during stress response [1]. High concentration of corticosterone causes neuronal atrophy in the hippocampus and cerebral cortex, and it also can lead to the pathogenesis of neurodegenerative diseases such as Alzheimer's disease or Parkinson's disease [31]. Excessive corticosterone level also can accelerate the inflammatory reactions by releasing pro-inflammatory cytokines including IL-1β, IL-6 and TNF-α [32]. In our experiment, these cytokine were considerably increased by whisker removal stress, especially in cerebral cortex, and pre-treatment with Myelophil significantly attenuates these alterations (Fig. 5-A to C). The whisker removal-induced considerable decreases of IFN-γ level was significantly ameliorated by pre-treatment with Myelophil (Fig. 5-D). The psycho-emotional stress is closely associated with immune dysfunctions, and IFN-γ, a vital cytokine of innate immunity, was known to be lowered in stress condition [33]. These changes of cytokine levels were generally correlated with those of gene expressions including IL-10 and iNOS (Fig. 6-A and B). Partial differences were observed between cerebral cortex and hippocampus, likely sensitive up-regulation of TNF-α in cerebral cortex and iNOS hippocampus after whisker removal (Fig. 6-A and B).

Myelophil has been developed based on the clinical experience and pharmacological theory of traditional Chinese medicine. Myelophil is composed of Astragali Radix and Salviae Radix which maintain homeostasis of *qi* and *blood* in the human body [14]. Other studies partially supported that the effects of Myelophil in our results. Salvianolic acid B and Astragaloside IV, a major compound of Salviae Radix and Astragali Radix respectively, reported the antioxidant and anti-inflammatory properties in brain tissue from other animal models [34], [35]. Our previous studies also showed the protective activities for brain tissue in immobilization and cold stress modes [15], [16].

In order to explore the molecular mechanism explaining above pharmacological actions of Myelophil, we herein investigated the activity of nuclear kappa-B (NF-κB), a key regulatory protein for inflammation. As pro-inflammatory cytokines IL-1β, IL-6 and TNF-α are main targets of transcription factor NF-κB during inflammatory reaction [36]. Pre-treatment with Myelophil dramatically inhibited the translocation of NF-κB into nucleolus, and these results were identically observed in both cerebral cortex and hippocampus (Fig. 7, Fig. S3-A to D).

We partially evidenced the anti-oxidative effects of Myelophil against brain injury using restraint and cold stress model [15], [16]. Those two models represent physical-dominant stress conditions, and conducted under the chronic stress status in mice. The current study is acute and psycho-emotional stress model, which oxidative injury was drastically induced in 12 hours. The present results additionally showed that Myelophil exerts antioxidant properties in cerebral cortex and hippocampus which are most vulnerable regions under severe stress status.

In summary, we provide experimental evidences that Myelophil efficiently protects brain tissue, especially cerebral cortex and hippocampus, from oxidative injury under psycho-emotional stress model. The underlying mechanisms may involve an enhancement of antioxidant properties and regulation of inflammatory proteins, especially via controlling NF-κB activation.

## Supporting Information

Figure S1 **Effects of Myelophil on the neuronal cell layer areas and 4-HNE signal density.** The neuronal cell areas in cerebral cortex (A) and hippocampal *cornus ammonis* (CA) 1 regions (B) were analyzed. The 4-HNE positive signal density was analyzed in cerebral cortex (C) and hioppocampal CA 1 region (D). Data are means ± standard deviations (n = 3). ^###^ *p*<0.001compared with the normal group; ^*^ *p*<0.05, ^**^ *p*<0.01 and ^***^ *p*<0.001 compared with the control group.
(TIF)

Figure S2 **Anti-apoptotic effects of Myelophil in brain tissue.** Cell death was analyzed in prefrontal cortex (A) and hippocampal *cornus ammonis* (CA) 1 regions (B) using TUNEL staining and observation under light microscopy (200× magnification, n = 3). The reference bar indicated 50 µm.
(TIF)

Figure S3 **Protein density analysis of western blot.** The protein densities of IκBα (cytosolic extract) and NF-κB (nuclear extract) in cerebral cortex (A and B) and in hippocampus (C and D) were determined. Data are means ± standard deviations (n = 4). ^###^ *p*<0.001compared with the normal group; ^*^ *p*<0.05, ^**^ *p*<0.01 and ^***^ *p*<0.001 compared with the control group.
(TIF)

Methods S1 **Determination of cell death signaling in brain tissues.** The Terminal deoxynucleotidyl transferase dUTP nick end labeling (TUNEL) assay was performed to detect apoptotic cells in brain tissue sections including prefrontal cortex and hippocampal cornus ammonis (CA) 1 regions using a commercial TUNEL Apoptosis Detection Kit (Millipore). Briefly, tissue sections were fixed in 10% formalin and embedded with paraffin. After deparaffinization and washing, the tissue specimens were incubated with Proteinase Katroomtem-perature for15 min. Then, 3% of H_2_O_2_ was applied to quench any remnant peroxidase. After several washes, the specimens were incubated with TdT-enzyme at 37°C for 90 min followed by anti-digoxigenin-peroxidase treatment for 60 min at room temperature. 3-Amino-9-ethylcarbazolewasusedasthe final chromogen during color development and the apoptotic cells were examined under a light microscope (200× magnification; Olympus, CenterValley, PA, USA) in randomly chosen fields.
(DOCX)
