# Supplementary figures and images for: Ethanolic Extract of Astragali Radix and Salviae Radix Prohibits Oxidative Brain Injury by Psycho-Emotional Stress in Whisker Removal Rat Model
Source: PLoS One. 2014 May 28;9(5):e98329. doi: 10.1371/journal.pone.0098329 (PMC4037216; doi:10.1371/journal.pone.0098329)

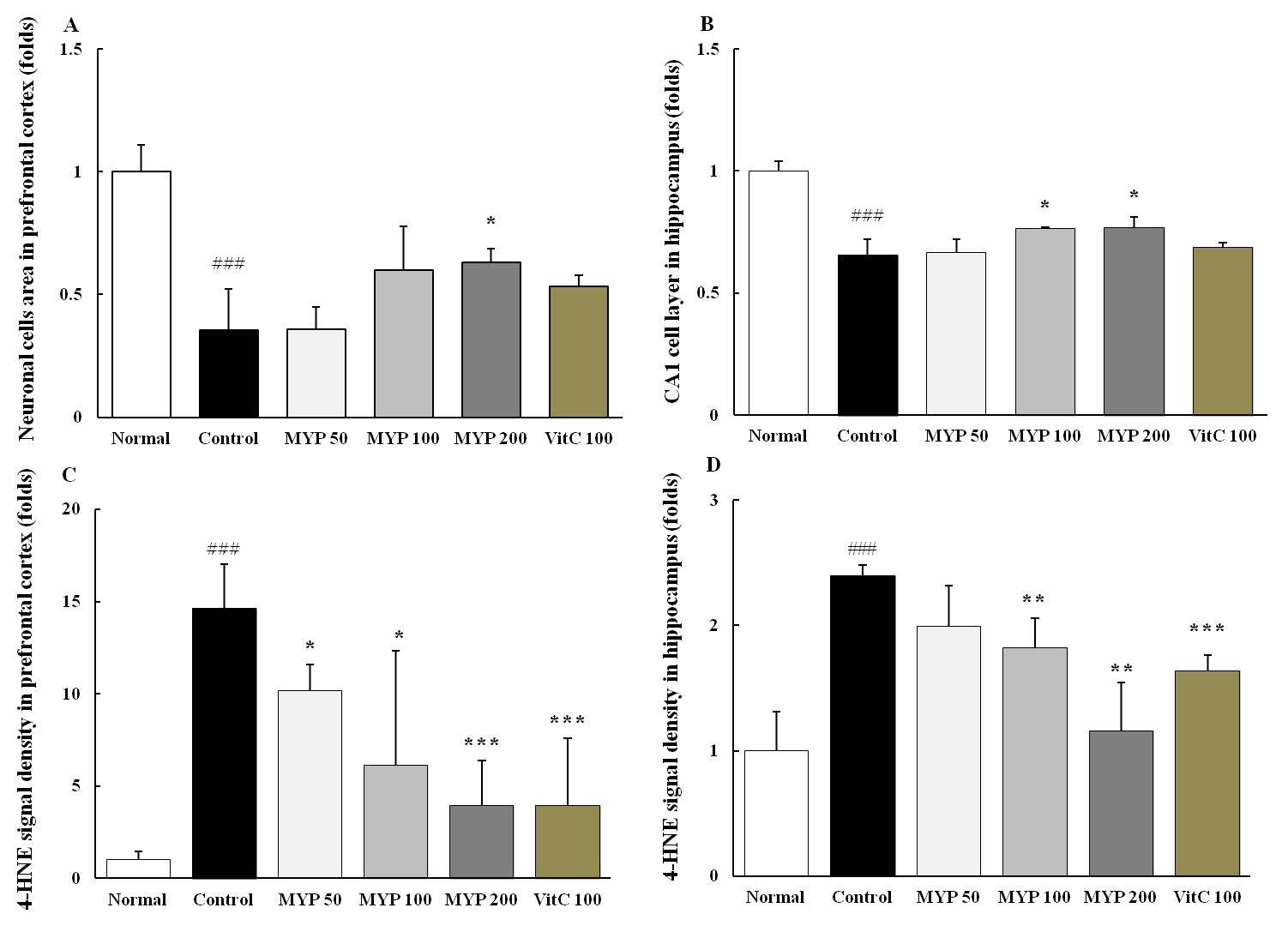

Supplement: Figure S1 — Effects of Myelophil on the neuronal cell layer areas and 4-HNE signal density. The neuronal cell areas in cerebral cortex (A) and hippocampal cornus ammonis (CA) 1 regions (B) were analyzed. The 4-HNE positive signal density was analyzed in cerebral cortex (C) and hioppocampal CA 1 region (D). Data are means ± standard deviations (n = 3). ### p<0.001compared with the normal group; * p<0.05, ** p<0.01 and *** p<0.001 compared with the control group. (TIF) [file pone.0098329.s001.tif]

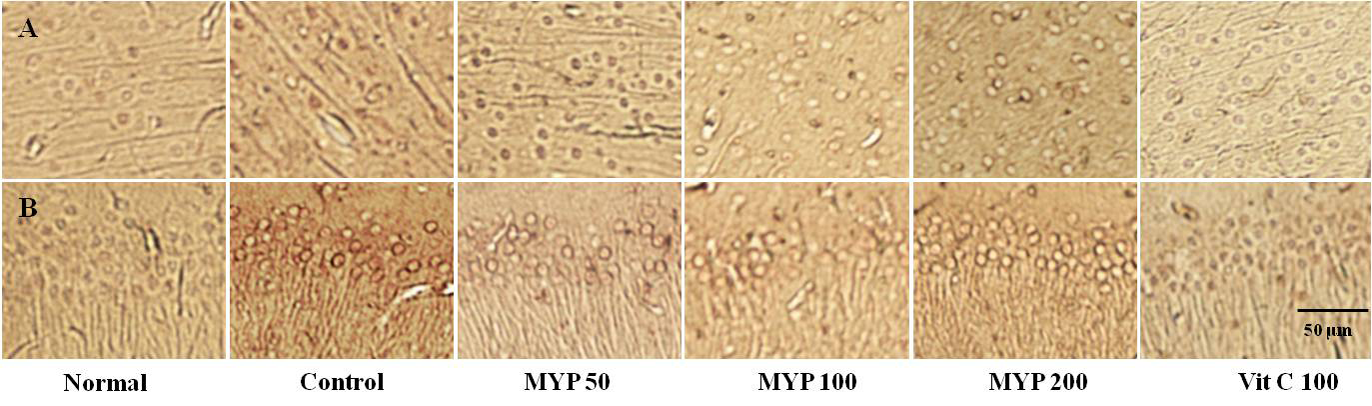

Supplement: Figure S2 — Anti-apoptotic effects of Myelophil in brain tissue. Cell death was analyzed in prefrontal cortex (A) and hippocampal cornus ammonis (CA) 1 regions (B) using TUNEL staining and observation under light microscopy (200× magnification, n = 3). The reference bar indicated 50 µm. (TIF) [file pone.0098329.s002.tif]

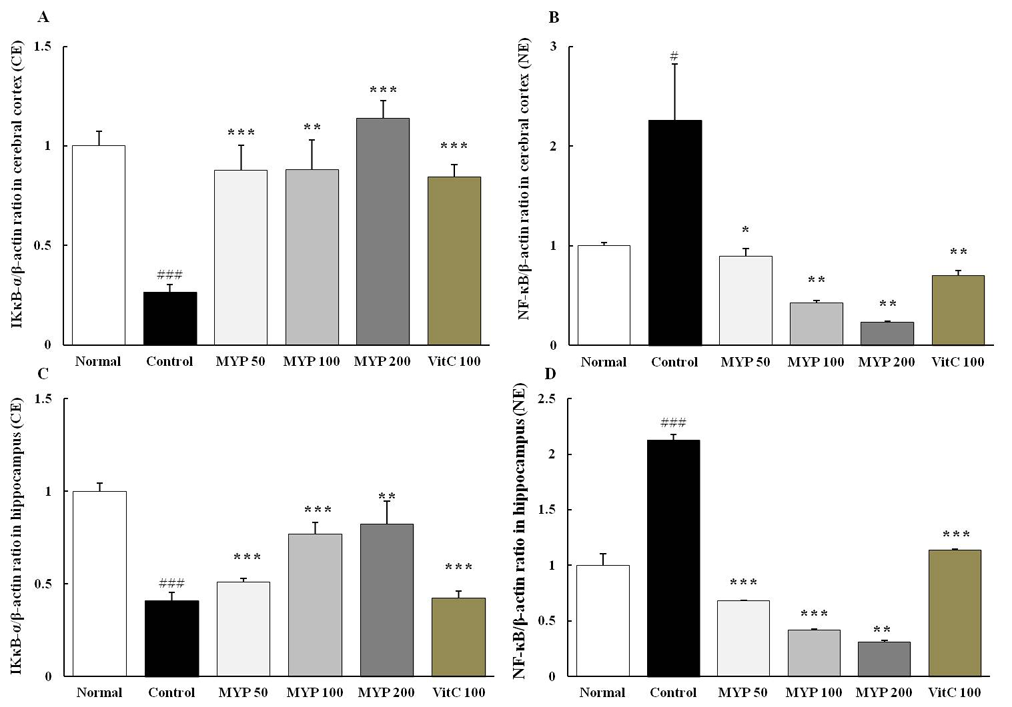

Supplement: Figure S3 — Protein density analysis of western blot. The protein densities of IκBα (cytosolic extract) and NF-κB (nuclear extract) in cerebral cortex (A and B) and in hippocampus (C and D) were determined. Data are means ± standard deviations (n = 4). ### p<0.001compared with the normal group; * p<0.05, ** p<0.01 and *** p<0.001 compared with the control group. (TIF) [file pone.0098329.s003.tif]
