# Supplementary material for: Ethanolic Extract of Astragali Radix and Salviae Radix Prohibits Oxidative Brain Injury by Psycho-Emotional Stress in Whisker Removal Rat Model
Source: PLoS One. 2014 May 28;9(5):e98329. doi: 10.1371/journal.pone.0098329 (PMC4037216; doi:10.1371/journal.pone.0098329)
Supplement: Methods S1 — Determination of cell death signaling in brain tissues. The Terminal deoxynucleotidyl transferase dUTP nick end labeling (TUNEL) assay was performed to detect apoptotic cells in brain tissue sections including prefrontal cortex and hippocampal cornus ammonis (CA) 1 regions using a commercial TUNEL Apoptosis Detection Kit (Millipore). Briefly, tissue sections were fixed in 10% formalin and embedded with paraffin. After deparaffinization and washing, the tissue specimens were incubated with Proteinase Katroomtem-perature for15 min. Then, 3% of H2O2 was applied to quench any remnant peroxidase. After several washes, the specimens were incubated with TdT-enzyme at 37°C for 90 min followed by anti-digoxigenin-peroxidase treatment for 60 min at room temperature. 3-Amino-9-ethylcarbazolewasusedasthe final chromogen during color development and the apoptotic cells were examined under a light microscope (200× magnification; Olympus, CenterValley, PA, USA) in randomly chosen fields. (DOCX) [file pone.0098329.s004.docx]

**Supporting Information Legends**

**Determination of cell death signaling in brain tissues**

The Terminal deoxynucleotidyl transferase dUTP nick end labeling (TUNEL) assay was performed to detect apoptotic cells in brain tissue sections including prefrontal cortex and hippocampal *cornus ammonis* (CA) 1 regions using a commercial TUNEL Apoptosis Detection Kit (Millipore). Briefly, tissue sections were fixed in 10% formalin and embedded with paraffin. After deparaffinization and washing, the tissue specimens were incubated with Proteinase Katroomtem-perature for15 min. Then, 3% of H_2_O_2_ was applied to quench any remnant peroxidase. After several washes, the specimens were incubated with TdT-enzyme at 37 °C for 90min followed by anti-digoxigenin-peroxidase treatment for 60min at room temperature. 3-Amino-9-ethylcarbazolewasusedasthe final chromogen during color development and the apoptotic cells were examined under a light microscope (200 × magnification; Olympus, CenterValley, PA, USA) in randomly chosen fields.

**Figure S1. Effects of Myelophil on the neuronal cell layer areas and 4-HNE signal density.** The neuronal cell areas in cerebral cortex (A) and hippocampal *cornus ammonis* (CA) 1 regions (B) were analyzed. The 4-HNE positive signal density was analyzed in cerebral cortex (C) and hioppocampal CA 1 region (D). Data are means ± standard deviations (n = 3). ^###^*p* < 0.001compared with the normal group; **^*^***p* < 0.05, ^**^*p* < 0.01 and ^***^*p* < 0.001 compared with the control group.

**Figure S2. Anti-apoptotic effects of Myelophil in brain tissue.** Cell death was analyzed in prefrontal cortex (A) and hippocampal *cornus ammonis* (CA) 1 regions (B) using TUNEL staining and observation under light microscopy (200 × magnification, n = 3). The reference bar indicated 50 μm.

**Figure S3. Protein density analysis of western blot.** The protein densities of IκBα (cytosolic extract) and NF-κB (nuclear extract) in cerebral cortex (A and B) and in hippocampus (C and D) were determined. Data are means ± standard deviations (n = 4). ^###^*p* < 0.001compared with the normal group; **^*^***p* < 0.05, ^**^*p* < 0.01 and ^***^*p* < 0.001 compared with the control group.
